# Effect of Dosage Reduction of Hypoglycemic Multidrug Regimens on the Incidences of Acute Glycemic Complications in People With Type 2 Diabetes Who Fast During Ramaḍān: A Randomized Controlled Trial

**DOI:** 10.3389/fendo.2021.613826

**Published:** 2021-07-07

**Authors:** Louay Y. Zaghlol, Amir F. Beirat, Justin Z. Amarin, Amro M. Hassoun Al Najar, Yazan Y. Hasan, Abdallah Qtaishat, Michael E. Tierney, Raja Y. Zaghlol, Ayman A. Zayed

**Affiliations:** ^1^ The University of Jordan School of Medicine, Amman, Jordan; ^2^ Hunter–New England Health, New Lambton, NSW, Australia; ^3^ Division of Internal Medicine, MedStar Georgetown University Hospital/Washington Hospital Center, Washington, DC, United States; ^4^ Department of Internal Medicine, The University of Jordan School of Medicine, Amman, Jordan

**Keywords:** type 2 diabetes mellitus, hypoglycemic agents, hypoglycemia, hyperglycemia, fasting

## Abstract

**Objective:**

We aimed to investigate the effect of dosage reduction of four hypoglycemic multidrug regimens on the incidences of acute glycemic complications in people with type 2 diabetes who fast during Ramaḍān.

**Methods:**

We conducted an open-label, parallel-group, randomized controlled trial at a tertiary care center in Amman, Jordan. We recruited adults with type 2 diabetes who expressed an intention to fast during Ramaḍān and were adherent to one of four regimens—namely: metformin and glimepiride; metformin and vildagliptin; metformin and insulin glargine U100; or, metformin, insulin glargine U100, and human regular insulin. We randomly assigned participants in a 2:1 ratio to low- or regular-dosage therapy. The primary outcomes were the incidences of hypoglycemia and hyperglycemia during the 29 days of Ramaḍān 2017, and the secondary outcomes were the incidences of diabetic ketoacidosis and hyperosmolar hyperglycemic state during the same period.

**Results:**

We randomly assigned 687 participants to low-dosage therapy (*n* = 458) or regular-dosage therapy (*n* = 229) and included 678 (452 and 226, respectively) in the final analysis. The incidence of hypoglycemia was lower in the low-dosage group compared with the regular-dosage group (19 [4.2%] vs. 52 [23.0%], respectively; OR, 0.15 [95% CI, 0.08–0.26]; *P* < 0.001). The incidence of hyperglycemia did not differ between the low- and regular-dosage groups (319 [70.6%] vs. 154 [68.1%], respectively; OR, 1.12 [95% CI, 0.79–1.58]; *P* = 0.5). No participants experienced diabetic ketoacidosis or hyperosmolar hyperglycemic state. Each 1% decrease in the baseline HbA_1c_ concentration was associated with a 19.9-fold (95% CI, 9.6–41.5; *P* < 0.001) increase in the odds of hypoglycemia, and each 1% increase in the baseline HbA_1c_ concentration was associated with a 15.7-fold (95% CI, 10.0–24.6; *P* < 0.001) increase in the odds of hyperglycemia.

**Conclusion:**

Dosage reduction decreases the incidence of hypoglycemia without a concomitant increase in the incidences of hyperglycemia, diabetic ketoacidosis, and hyperosmolar hyperglycemic state in people with type 2 diabetes who fast during Ramaḍān.

**Clinical Trial Registration:**

www.ClinicalTrials.gov, identifier NCT04237493.

## Introduction

Every lunar year, during the month of Ramaḍān, Muslims abstain from food and drink between dawn and nightfall. People who fast usually consume a meal after nightfall (ifār), another before dawn (suūr), and multiple snacks in between (1). Muslims with a chronic disease, such as type 2 diabetes, are exempt from fasting, but many choose to fast despite the health risks. According to a population-based study, 79% of Muslims with type 2 diabetes (~80 million people worldwide) observe the fast (2). People with type 2 diabetes who fast during Ramaḍān are at increased risk of acute glycemic complications—namely: hypoglycemia, hyperglycemia, diabetic ketoacidosis, and hyperosmolar hyperglycemic state (3).

One strategy to prevent acute glycemic complications is dosage reduction of hypoglycemic drug therapy (4). Ideally, dosage reduction should produce a decrease in the incidence of hypoglycemia without a concomitant increase in the incidence of hyperglycemia. Currently, evidence for or against the safety and efficacy of dosage reduction in people with type 2 diabetes who fast during Ramaḍān is scarce (1, 4, 5). Some randomized controlled trials (RCTs) have investigated the effect of dosage reduction of one or two hypoglycemic drugs on the incidence of hypoglycemia in people with type 2 diabetes who fast during Ramaḍān (6–8). However, many common hypoglycemic multidrug regimens have not been investigated, and the incidences of other important acute glycemic complications have not been measured.

Therefore, we aimed to investigate the effect of dosage reduction of four hypoglycemic multidrug regimens on the incidences of acute glycemic complications in people with type 2 diabetes who fast during Ramaḍān.

## Materials and Methods

### Trial Design and Participants

We conducted an open-label, parallel-group, randomized controlled trial at a tertiary care center in Amman, Jordan. We recruited people with type 2 diabetes from the endocrinology outpatient clinics between February and April 2017. Eligible participants were adults (≥18 years) who expressed an intention to fast during Ramaḍān 2017 (between May 27 and June 24, 2017) and were adherent to one of four hypoglycemic multidrug regimens for at least the past three months. The four regimens are outlined in **Table 1**. We selected these four regimens because they were the most commonly used by people who attended our clinics during the study period. Newer drug classes, such as sodium–glucose cotransporter 2 (SGLT2) inhibitors and glucagon-like peptide-1 (GLP-1) receptor agonists, were not commonly used by our patients. We defined treatment adherence as use of ≥80% of prescribed medication (by pill and/or vial count).

**Table 1:** The hypoglycemic multidrug regimens used during Ramaḍān by our participants, who we randomly allocated to low- or regular-dosage therapy (*N* = 678 participants).

Multidrug Regimens	Regular Dosage	Low Dosage
Metformin	850 mg BID^a^	850 mg BID^a^
Glimepiride	2 or 4 mg OD^b^ (100% of the pre-Ramaḍān dose)	1.5 or 3 mg OD^b^ (75% of the pre-Ramaḍān dose)
Metformin	850 mg BID^a^	850 mg BID^a^
Vildagliptin	50 mg BID^a^	50 mg OD^b^
Metformin	850 mg BID^a^	850 mg BID^a^
Insulin Glargine U100	11–44 units^c^ (100% of the pre-Ramaḍān dose)	9–33 units^c^ (75% of the pre-Ramaḍān dose)
Metformin	850 mg BID^a^	850 mg BID^a^
Insulin Glargine U100	8–52 units^c^ (100% of the pre-Ramaḍān dose)	6–39 units^c^ (75% of the pre-Ramaḍān dose)
Human Regular Insulin	12–40 units^d^, 7–28 units^e^ (100% of the pre-Ramaḍān doses)^f^	15–33 units^d^, 6–18 units^e^ (75% of the pre-Ramaḍān doses)^f^

a To be taken with ifār and with suūr.

b To be taken with ifār.

c To be injected at 10 P.M.

d To be injected before ifār.

e To be injected before suūr.

f We did not prescribe the pre-Ramaḍān third injection during Ramaḍān.

We excluded people with type 1 diabetes, latent autoimmune diabetes in adults, secondary diabetes, or autonomic neuropathy. In addition, we excluded pregnant, postpartum, breastfeeding, and eumenorrheic women. Pregnant, postpartum, and breastfeeding women are exempt from fasting, and eumenorrheic women do not fast during menstruation. We also excluded participants who experienced diabetic ketoacidosis or hyperosmolar hyperglycemic state within three months and those who received niacin or corticosteroids within one month. In addition, we excluded participants with a baseline hemoglobin A_1c_ (HbA_1c_) concentration below 6.0% (42 mmol/mol) or exceeding 11.0% (97 mmol/mol). Finally, we reviewed the medical records and excluded participants who had a history of recurrent hypoglycemia, hypoglycemia unawareness, chronic renal insufficiency (stages IV or V), liver cirrhosis, uncontrolled epilepsy, depressive disorder, bipolar disorder, psychotic disorder, or cognitive dysfunction.

### Procedures

A research assistant, who was otherwise not involved in the study, generated the allocation sequence and enclosed the assignments in sequentially numbered, opaque, sealed envelopes. We enrolled participants and randomly assigned them (2:1 ratio) to low- or regular-dosage therapy. We stratified randomization by treatment regimen and used unequal allocation to obtain more safety data for low-dosage therapy. We determined the extent of dosage reduction (**Table 1**) based on our experience from a previous study (9).

We asked participants to attend a clinic visit before Ramaḍān and another after Ramaḍān. During the pre-Ramaḍān clinic visit, we prescribed the treatment dosage to be taken during Ramaḍān and asked participants to self-monitor their blood glucose concentration four times a day—namely, before suūr (the meal before dawn), at noon, before ifār (the meal after nightfall), and two hours after ifār—and if they developed any symptoms of hypoglycemia or hyperglycemia. We also offered participants the option to continue using their own glucometer and test strips if the device met the ISO 15197:2013 standard. Otherwise, we offered participants a new glucometer and test strips. We advised participants to break the fast and seek medical attention if they experienced any symptom of hypoglycemia or hyperglycemia or recorded a blood glucose concentration ≤70 mg/dL (3.9 mmol/L). Finally, we recorded nine baseline characteristics—namely: age, biologic sex, years since diagnosis, baseline HbA_1c_ concentration, body mass index (BMI), polypharmacy, β1-blocker use, angiotensin-converting enzyme (ACE) inhibitor use, and number of comorbidities. We classified BMI according to the standard World Health Organization classification system (10). We defined polypharmacy as the concurrent, long-term use of five or more drugs (11). During the post-Ramaḍān clinic visit, we assessed the treatment and fasting adherence of participants, and we measured the outcomes using interviews, chart reviews and glucometer readings. We defined fasting adherence as observance of the full Ramaḍān fast excluding per-protocol breaking of the fast.

### Outcomes

The primary outcomes were the incidences of hypoglycemia and hyperglycemia during the 29 days of Ramaḍān 2017. We identified and classified hypoglycemia based on the definitions of the International Hypoglycaemia Study Group (12). Briefly, level 1 hypoglycemia corresponds to a blood glucose concentration ≤70 mg/dL (3.9 mmol/L), level 2 hypoglycemia corresponds to a blood glucose concentration ≤54 mg/dL (3.0 mmol/L), and level 3 hypoglycemia denotes hypoglycemia with severe cognitive impairment requiring external assistance for recovery. We defined hyperglycemia as a blood glucose concentration ≥200 mg/dL (11.1 mmol/L). The secondary outcomes were the incidences of diabetic ketoacidosis and hyperosmolar hyperglycemic state (defined according to a consensus report by the American Diabetes Association) during the same period (13).

### Statistical Methods

We used R (version 4.0.2) to perform data analysis. To calculate the minimum number of participants required, we anticipated incidences of hypoglycemia of 7.5% and 15% in the low- and regular-dosage groups, respectively, for an overall incidence of 10% (14). The minimum target was 597 participants (α = 0.05, β = 0.2). We summarized categorical data as *n* (%) and continuous data as mean ± standard deviation. To measure the association between treatment dosage and the level of hypoglycemia, we used Pearson’s *χ* ^2^ test. To compare the distribution of the number of episodes of hyperglycemia between the low- and regular-dosage groups, we used the Mann–Whitney *U* test. We used Pearson’s *χ* ^2^ test to compare the primary outcomes between the low- and regular-dosage groups and binary logistic regression to model the primary outcomes as functions of treatment dosage, treatment regimen, age, biologic sex, years since diagnosis, BMI, baseline HbA_1c_ concentration, and polypharmacy. We handled missing data according to the recommendations of Jakobsen et al. (15). We used the Benjamini–Hochberg method to correct for multiple testing and interpreted values of *P* < 0.05 (two-tailed) to indicate statistical significance.

### Ethics

The Institutional Review Board of the Jordan University Hospital approved the trial (2017/35). We did not offer a financial incentive for participation, and all participants provided written informed consent. The trial is registered with ClinicalTrials.gov (NCT04237493).

## Results

**Figure 1:**
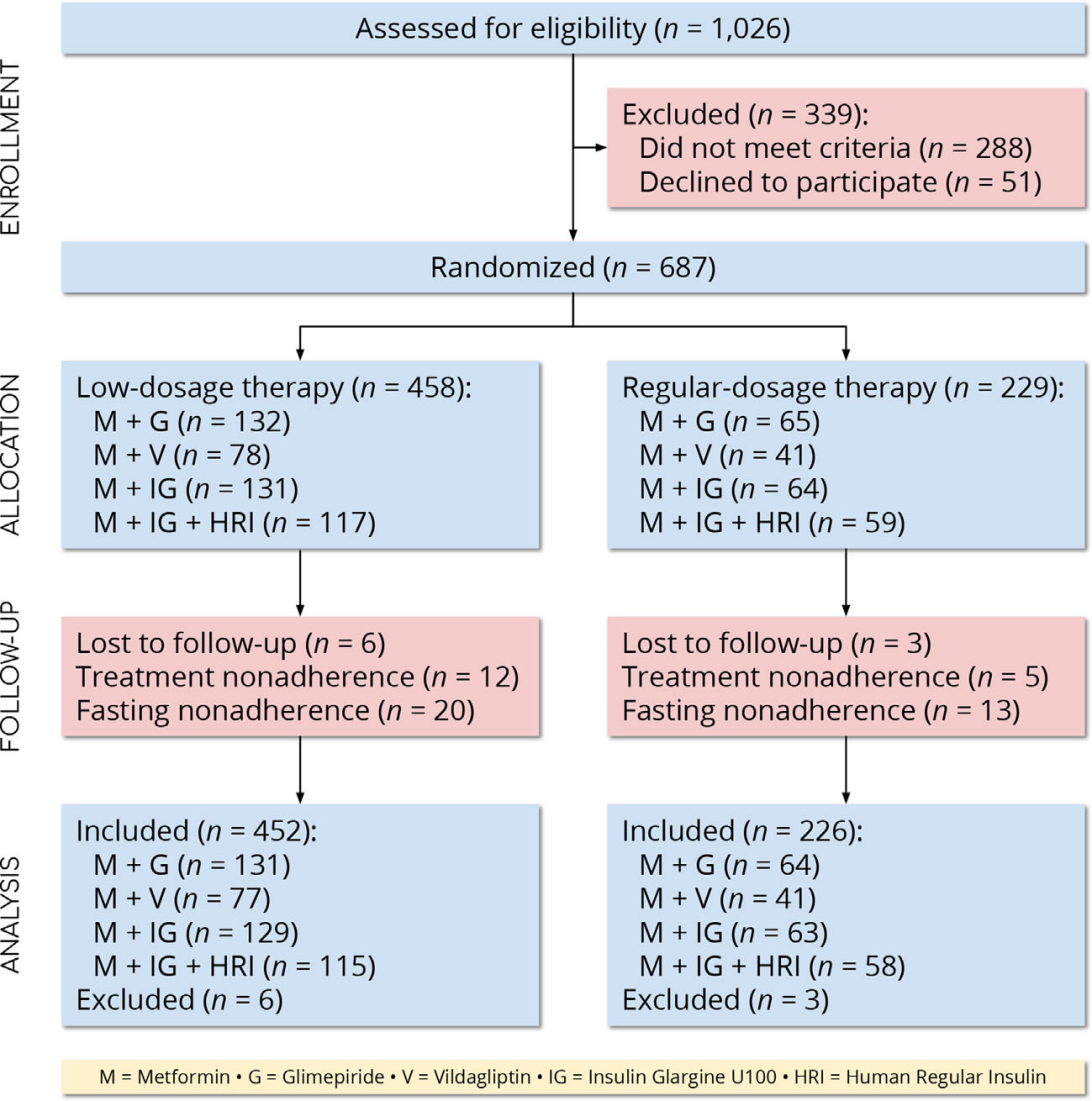
CONSORT flow diagram. (*N* = 678 participants).

We screened 1,026 people for eligibility and 687 (67.0%) met the eligibility criteria. We excluded nine participants (1.3%) with missing outcomes and included the remaining 678 (98.7%) in the final analysis (**Figure 1**). The numbers of participants in the low- and regular-dosage groups were 452 (66.7%) and 226 (33.3%), respectively. In total, 195 participants (28.8%) were on metformin and glimepiride; 118 (17.4%) were on metformin and vildagliptin; 192 (28.3%) were on metformin and insulin glargine U100; and, 173 (25.5%) were on metformin, insulin glargine U100, and human regular insulin. We report the baseline characteristics of the study population in **Table 2** (by-dosage and total) and **Supplementary Table 1** (by-regimen and by-dosage).

**Table 2:** Baseline characteristics of the study population (*N* = 678 participants).

Characteristics	Total, *N* = 678	Low Dosage, *n* = 452	Regular Dosage, *n* = 226
Age (years)	58.0 ± 9.8	58.4 ± 9.7	57.4 ± 9.8
Sex			
* Male*	336 (49.6%)	222 (49.1%)	114 (50.4%)
*Female*	342 (50.4%)	230 (50.9%)	112 (49.6%)
Body mass index			
*Normal weight*	15 (2.2%)	11 (2.4%)	4 (1.8%)
*Overweight*	568 (83.8%)	375 (83.0%)	193 (85.4%)
*Obese (class I)*	95 (14.0%)	66 (14.6%)	29 (12.8%)
Years since diagnosis	8.2 ± 5.0	8.3 ± 5.2	8.0 ± 4.8
Baseline HbA_1c_ (%)	8.1 ± 0.9	8.1 ± 0.9	8.1 ± 1.0
Polypharmacy			
*No*	584 (86.1%)	385 (85.2%)	199 (88.1%)
*Yes*	94 (13.9%)	67 (14.8%)	27 (11.9%)
β1-blocker use			
*No*	579 (85.4%)	383 (84.7%)	196 (86.7%)
*Yes*	99 (14.6%)	69 (15.3%)	30 (13.3%)
ACE inhibitor use			
*No*	627 (92.5%)	414 (91.6%)	213 (94.2%)
*Yes*	51 (7.5%)	38 (8.4%)	13 (5.8%)
Number of comorbidities	2.4 ± 1.3	2.4 ± 1.3	2.3 ± 1.3

Data are n (%) or mean ± standard deviation.

ACE, angiotensin-converting enzyme.

We report the outcomes of the study population in **Table 3**. Hypoglycemia occurred in 71 participants (10.5%), with a lower incidence in the low-dosage group compared with the regular-dosage group (19 [4.2%] vs. 52 [23.0%], respectively; *P* < 0.001). The difference in incidences was consistent across all four treatment regimens. Of the participants in the low-dosage group, 14 (73.7%) experienced level 1 hypoglycemia and the remaining five (26.3%) experienced level 2 hypoglycemia. Of the participants in the regular-dosage group, 32 (61.5%) experienced level 1 hypoglycemia and the remaining 20 (38.5%) experienced level 2 hypoglycemia. No participants experienced level 1 or 2 hypoglycemia more than once, and no participants experienced level 3 hypoglycemia. In participants who experienced level 1 or 2 hypoglycemia, treatment dosage was not associated with the level of hypoglycemia (*P* = 0.3). Hyperglycemia occurred in 473 participants (69.8%), with no statistically significant difference in the incidences between the low- and regular-dosage groups (319 [70.6%] vs. 154 [68.1%], respectively; *P* = 0.5). The distribution of the number of episodes of hyperglycemia did not differ between the groups (*P* = 0.5), and the incidences of hyperglycemia were not statistically significantly different between the groups across all four treatment regimens. No participants experienced diabetic ketoacidosis or hyperosmolar hyperglycemic state.

**Table 3:** Outcomes of the study population (*N* = 678 participants).

Outcomes	Total, *N* = 678	Low Dosage, *n* = 452	Regular Dosage, *n* = 226	Odds Ratio (95% CI)	*P* value
Incidence of hypoglycemia					
*M + G*	24 (12.3%)	7 (5.3%)	17 (26.6%)	0.16 (0.06–0.40)	<0.001
*M + V*	7 (5.9%)	1 (1.3%)	6 (14.6%)	0.08 (0.01–0.66)	0.004
*M + IG*	18 (9.4%)	5 (3.9%)	13 (20.6%)	0.16 (0.05–0.46)	<0.001
*M + IG + HRI*	22 (12.7%)	6 (5.2%)	16 (27.6%)	0.14 (0.05–0.39)	<0.001
*Overall*	71 (10.5%)	19 (4.2%)	52 (23.0%)	0.15 (0.08–0.26)	<0.001
Incidence of hyperglycemia					
*M + G*	128 (65.6%)	90 (68.7%)	38 (59.4%)	1.50 (0.81–2.79)	0.4
*M + V*	89 (75.4%)	59 (76.6%)	30 (73.2%)	1.20 (0.50–2.87)	0.7
*M + IG*	145 (75.5%)	100 (77.5%)	45 (71.4%)	1.38 (0.70–2.74)	0.5
*M + IG + HRI*	111 (64.2%)	70 (60.9%)	41 (70.7%)	0.64 (0.33–1.27)	0.4
*Overall*	473 (69.8%)	319 (70.6%)	154 (68.1%)	1.12 (0.79–1.58)	0.5
Incidence of diabetic ketoacidosis					
*Overall*	0 (0%)	0 (0%)	0 (0%)		
Incidence of hyperosmolar hyperglycemic state					
*Overall*	0 (0%)	0 (0%)	0 (0%)		

Data are n (%).

M, metformin; G, glimepiride; V, vildagliptin; IG, insulin glargine U100; HRI, human regular insulin.

In multivariable analysis, treatment dosage and baseline HbA_1c_ concentration were independent predictors of the incidence of hypoglycemia (**Table 4**). Each 1% (11 mmol/mol) decrease in the baseline HbA_1c_ concentration predicted a 19.9-fold increase in the odds of hypoglycemia. Notably, treatment dosage was not an independent predictor of the incidence of hyperglycemia. However, the odds of hyperglycemia was higher in participants who were on metformin and vildagliptin compared with participants who were on metformin and glimepiride or metformin, insulin glargine U100, and human regular insulin. In addition, age, years since diagnosis, and baseline HbA_1c_ concentration were also independent predictors of the incidence of hyperglycemia (**Table 4**). Each 1% (11 mmol/mol) increase in the baseline HbA_1c_ concentration predicted a 15.7-fold increase in the odds of hyperglycemia.

**Table 4:** Predictors of the incidences of hypoglycemia and hyperglycemia (*N* = 678 participants).

Predictors	Incidence of hypoglycemia		Incidence of hyperglycemia	
	Odds Ratio (95% CI)	*P* value	Odds Ratio (95% CI)	*P* value
Treatment dosage	0.075 (0.037–0.151)	<0.001	1.18 (0.74–1.88)	0.5
Treatment regimen				
*M + G vs. M + V*	2.47 (0.80–7.65)	0.4	0.35 (0.17–0.71)	0.01
*M + G vs. M + IG*	1.05 (0.45–2.45)	1	0.68 (0.38–1.22)	0.3
*M + G vs. M + IG + HRI*	0.61 (0.27–1.41)	0.5	1.12 (0.61–2.04)	0.7
*M + V vs. M + IG*	0.43 (0.14–1.34)	0.4	1.95 (0.94–4.04)	0.2
*M + V vs. M + IG + HRI*	0.25 (0.08–0.75)	0.06	3.19 (1.56–6.51)	0.005
*M + IG vs. M + IG + HRI*	0.58 (0.25–1.37)	0.5	1.64 (0.87–3.08)	0.2
Age (years)	1.03 (0.98–1.08)	0.5	1.06 (1.02–1.10)	0.005
Sex	1.12 (0.59–2.11)	0.9	1.16 (0.75–1.81)	0.5
Years since diagnosis	0.97 (0.88–1.08)	0.9	0.81 (0.75–0.87)	<0.001
BMI (kg m^-2^)	1.00 (0.83–1.21)	1	0.93 (0.82–1.06)	0.4
Baseline HbA_1c_ (%)	0.050 (0.024–0.104)	<0.001	15.7 (10.0–24.6)	<0.001
Polypharmacy	1.26 (0.43–3.70)	0.9	1.50 (0.70–3.24)	0.4

M, metformin; G, glimepiride; V, vildagliptin; IG, insulin glargine U100; HRI, human regular insulin.

## Discussion

We conducted the first randomized controlled trial to investigate the effect of dosage reduction of four hypoglycemic multidrug regimens on the incidences of acute glycemic complications in people with type 2 diabetes who fast during Ramaḍān. We found that: (a) Dosage reduction was associated with a decrease in the incidence of hypoglycemia without a concomitant increase in the incidence of hyperglycemia; (b) Both effects were consistent across people on any one of four hypoglycemic multidrug regimens—namely: metformin and glimepiride; metformin and vildagliptin; metformin and insulin glargine U100; and, metformin, insulin glargine U100, and human regular insulin; (c) No participants experienced diabetic ketoacidosis or hyperosmolar hyperglycemic state, which suggests that dosage reduction is safe; and, (d) An increase in the baseline HbA_1c_ concentration was independently associated with an increase in the odds of hyperglycemia but a decrease in the odds of hypoglycemia during Ramaḍān.

The incidence of hypoglycemia during Ramaḍān varies widely between different populations. Jabbar et al. reported that the incidence of hypoglycemia during Ramaḍān ranged from 4% in the Middle East to 13% in North Africa (16). In our sample, the incidence of hypoglycemia was 10.5% and ranged from 4.2% in the low-dosage group to 23.0% in the regular-dosage group. The difference in incidences was consistent across all four treatment regimens. For instance, we found that 25% dosage reduction of glimepiride was associated with a decrease in the incidence of hypoglycemia. In contrast, Belkhadir et al. reported that 25% dosage reduction of glyburide (another sulfonylurea compound) monotherapy was not associated with a decrease in the incidence of hypoglycemia during Ramaḍān (6). However, they did not identify episodes of hypoglycemia based on blood glucose concentrations. Rather, they rated the episodes on a “six point scale”, so the incidence of hypoglycemia they reported may be an underestimate.

Likewise, we found that dosage reduction of vildagliptin (in dual therapy with metformin) was associated with a decrease in the incidence of hypoglycemia. Current practice guidelines do not recommend dosage reduction of dipeptidyl-peptidase IV inhibitors in people with type 2 diabetes who fast during Ramaḍān (1, 4, 5). The recommendation is based on the finding that dipeptidyl-peptidase IV inhibitors are associated with a low risk of hypoglycemia in monotherapy and dual therapy with metformin (4, 17). In addition, Bosi et al. showed that the incidence of hypoglycemia does not differ between patients on metformin and 50 mg vildagliptin daily and those on metformin and 100 mg vildagliptin daily (18). The foregoing results seem likely to apply to people with type 2 diabetes who fast during Ramaḍān because dipeptidyl-peptidase IV inhibitors stimulate insulin secretion in a glucose-dependent manner (19). However, in our clinical experience, we observed that the incidence of hypoglycemia increases in people on metformin and 100 mg vildagliptin daily during Ramaḍān compared with the months before Ramaḍān. In support, Halimi et al. reported that the incidence of confirmed hypoglycemia in people on metformin and vildagliptin who fast during Ramaḍān was 24% (20). The discrepancy between these results remains unclear but we speculate that the increased incidence of hypoglycemia in people on metformin and 100 mg vildagliptin daily during Ramaḍān may be related to interindividual variability in the glucagon response to hypoglycemia (21, 22).

We also found that dosage reduction was not associated with hyperglycemia (overall and across all treatment regimens). Notably, the odds of hyperglycemia was higher in participants who were on metformin and vildagliptin compared with two other regimens (metformin and glimepiride; metformin, insulin glargine U100, and human regular insulin) independent of treatment dosage. To our knowledge, the effects of these regimens on the incidence of hyperglycemia in patients with type 2 diabetes who fast during Ramaḍān have not been compared in head-to-head trials. However, we speculate that the glucose-lowering effects of sulfonylureas and insulins are more potent than dipeptidyl-peptidase IV inhibitors during prolonged fasting because the latter stimulate insulin secretion in a glucose-dependent manner while the former are glucose-independent (19). Overall, the incidence of hyperglycemia in people with type 2 diabetes who fast during Ramaḍān is not well-established; compared with hypoglycemia, hyperglycemia is less often an outcome of interest. According to the EPIDIAR study—a population-based study of 11,173 people with type 2 diabetes—the incidence of hyperglycemia is higher in people who fast (2). However, according to the DAR–MENA T2DM study—a prospective, observational study of 1,749 people with type 2 diabetes—the incidence of hyperglycemia is lower in people who fast (14).

No participants experienced diabetic ketoacidosis or hyperosmolar hyperglycemic state, which supports the safety of dosage reduction. We observed similar results in a previous study on another group of people with type 2 diabetes who fast during Ramaḍān (9). Some authors have suggested that people with diabetes who fast during Ramaḍān are at increased risk of diabetic ketoacidosis (23). The suggested reasons are insulin insufficiency associated with excess counterregulatory hormones, excessive dosage reduction of hypoglycemic drugs, and compensatory overeating during the nighttime. However, the reasons are unsubstantiated, and a recent review showed that the risk of diabetic ketoacidosis is not higher in people who fast (24).

We also found that people with a higher baseline HbA_1c_ concentration were less likely to experience hypoglycemia and more likely to experience hyperglycemia. In support, Loke et al. reported that an HbA_1c_ concentration less than 8% is a risk factor for hypoglycemia during Ramaḍān (25). Therefore, people with well-controlled type 2 diabetes who fast during Ramaḍān require closer monitoring and may benefit from dosage reduction beyond what we have proposed.

Our study has some limitations. First, we recruited participants from a single center by consecutive sampling. Therefore, our conclusions may not be generalizable. However, our center provides tertiary care to people from all the governorates of Jordan, so our conclusions may apply to the general population of Jordan. Notably, the incidence of hypoglycemia in Ramaḍān varies widely between the populations of different countries, and so our investigation must be replicated in other populations (16). Second, glycemic control during Ramaḍān is an important outcome that we did not measure. Unlike HbA_1c_, serum fructosamine is a suitable marker of short-term glycemic control (less than one month). However, we did not have access to the test. In addition, our methods may be improved by the use of continuous glucose monitoring. We recommend the use of serum fructosamine and continuous glucose monitoring in future investigations of people with diabetes who fast during Ramaḍān. Third, we did not assess participants for β-cell failure. However, we anticipate that the likelihood of β-cell failure in our participants is low because the mean duration of diabetes was 8.2 years since diagnosis, which is relatively short. According to the Veterans Affairs Diabetes Trial, the mean blood C-peptide—a marker of β-cell failure—in patients with type 2 diabetes of 13–15 years duration was ~0.7 nmol/L, which is considerably higher than the cutoff value (<0.2 nmol/L) for β-cell failure (26).

In conclusion, dosage reduction decreases the incidence of hypoglycemia without a concomitant increase in the incidences of hyperglycemia, diabetic ketoacidosis, and hyperosmolar hyperglycemic state in people with type 2 diabetes who fast during Ramaḍān.

## Data Availability Statement

The raw data supporting the conclusions of this article will be made available by the authors, without undue reservation.

## Ethics Statement

The studies involving human participants were reviewed and approved by Institutional Review Board of the Jordan University Hospital. The patients/participants provided their written informed consent to participate in this study.

## Author Contributions

AZ and LZ designed the study. LZ, AB, AH, YH, and AQ performed the literature search. AZ conducted the trial. AZ and AB collected the data. AZ, LZ, JA, and AB interpreted the data. JA performed data analysis. JA, LZ, and AZ wrote the report, and RZ and MT reviewed the report. All authors contributed to the article and approved the submitted version.

## Conflict of Interest

The authors declare that the research was conducted in the absence of any commercial or financial relationships that could be construed as a potential conflict of interest.
