# Supplementary material for: Effect of Dosage Reduction of Hypoglycemic Multidrug Regimens on the Incidences of Acute Glycemic Complications in People With Type 2 Diabetes Who Fast During Ramaḍān: A Randomized Controlled Trial
Source: Front Endocrinol (Lausanne). 2021 Jul 7;12:613826. doi: 10.3389/fendo.2021.613826 (PMC8294055; doi:10.3389/fendo.2021.613826)
Supplement: Supplementary file 1 [file Table_1.docx]

Supplementary Material

**Supplementary Table 1.** Baseline characteristics of the study population, stratified by regimen and dosage (*N* = 678 participants).

| **Characteristics** | **Metformin and glimepiride, *n*=195** | | **Metformin and vildagliptin, *n*=118** | | **Metformin and insulin glargine U100, *n*=192** | | **Metformin, insulin glargine U100, and human regular insulin, *n*=173** | |
| --- | --- | --- | --- | --- | --- | --- | --- | --- |
|  | **Low Dosage, *n*=131** | **Regular Dosage, *n*=64** | **Low Dosage, *n*=77** | **Regular Dosage, *n*=41** | **Low Dosage, *n*=129** | **Regular Dosage, *n*=63** | **Low Dosage, *n*=115** | **Regular Dosage, *n*=58** |
| Age (years) | 57.0 ± 9.5 | 57.2 ± 10.1 | 59.6 ± 8.8 | 57.4 ± 9.9 | 57.6 ± 9.5 | 58.0 ± 9.6 | 59.9 ± 10.6 | 57.1 ± 9.8 |
| Sex |  |  |  |  |  |  |  |  |
| *Male* | 65 (49.6%) | 29 (45.3%) | 41 (53.2%) | 24 (58.5%) | 61 (47.3%) | 32 (50.8%) | 55 (47.8%) | 29 (50.0%) |
| *Female* | 66 (50.4%) | 35 (54.7%) | 36 (46.8%) | 17 (41.5%) | 68 (52.7%) | 31 (49.2%) | 60 (52.2%) | 29 (50.0%) |
| BMI |  |  |  |  |  |  |  |  |
| *Normal weight* | 6 (4.6%) | 3 (4.7%) | 0 (0.0%) | 0 (0.0%) | 1 (0.8%) | 1 (1.6%) | 4 (3.5%) | 0 (0.0%) |
| *Overweight* | 107 (81.7%) | 50 (78.1%) | 64 (83.1%) | 38 (92.7%) | 114 (88.4%) | 54 (85.7%) | 90 (78.3%) | 51 (87.9%) |
| *Obese (class I)* | 18 (13.7%) | 11 (17.2%) | 13 (16.9%) | 3 (7.3%) | 14 (10.9%) | 8 (12.7%) | 21 (18.3%) | 7 (12.1%) |
| Years since diagnosis | 6.8 ± 4.0 | 7.5 ± 4.9 | 10.4 ± 5.0 | 9.5 ± 5.5 | 6.4 ± 4.0 | 7.9 ± 4.1 | 10.7 ± 6.1 | 7.5 ± 4.8 |
| Baseline HbA_1c_ (%) | 8.0 ± 0.9 | 8.0 ± 1.2 | 8.1 ± 0.8 | 8.1 ± 0.9 | 8.1 ± 0.9 | 8.1 ± 0.9 | 8.1 ± 0.9 | 8.1 ± 0.9 |
| Polypharmacy |  |  |  |  |  |  |  |  |
| *No* | 118 (90.1%) | 56 (87.5%) | 63 (81.8%) | 37 (90.2%) | 111 (86.0%) | 54 (85.7%) | 93 (80.9%) | 52 (89.7%) |
| *Yes* | 13 (9.9%) | 8 (12.5%) | 14 (18.2%) | 4 (9.8%) | 18 (14.0%) | 9 (14.3%) | 22 (19.1%) | 6 (10.3%) |
| β1-blocker use |  |  |  |  |  |  |  |  |
| *No* | 119 (90.8%) | 58 (90.6%) | 62 (80.5%) | 35 (85.4%) | 106 (82.2%) | 53 (84.1%) | 96 (83.5%) | 50 (86.2%) |
| *Yes* | 12 (9.2%) | 6 (9.4%) | 15 (19.5%) | 6 (14.6%) | 23 (17.8%) | 10 (15.9%) | 19 (16.5%) | 8 (13.8%) |
| ACE inhibitor use |  |  |  |  |  |  |  |  |
| *No* | 125 (95.4%) | 61 (95.3%) | 69 (89.6%) | 38 (92.7%) | 115 (89.1%) | 58 (92.1%) | 105 (91.3%) | 56 (96.6%) |
| *Yes* | 6 (4.6%) | 3 (4.7%) | 8 (10.4%) | 3 (7.3%) | 14 (10.9%) | 5 (7.9%) | 10 (8.7%) | 2 (3.4%) |
| Number of comorbidities | 2.5 ± 1.3 | 2.6 ± 1.4 | 2.7 ± 1.3 | 2.1 ± 1.2 | 2.2 ± 1.2 | 2.3 ± 1.3 | 2.4 ± 1.4 | 2.0 ± 1.2 |

Data are *n* (%) or mean ± standard deviation.

ACE, angiotensin-converting enzyme.
